# The role of brachytherapy in liver metastases from colorectal cancer

**DOI:** 10.3389/fimmu.2025.1641533

**Published:** 2025-08-07

**Authors:** Paweł Cisek, Izabela Kordzińska-Cisek, Ludmiła Grzybowska-Szatkowska

**Affiliations:** ^1^ Radiotherapy Department, Medical University of Lublin, Lublin, Poland; ^2^ Oncology Department, St. John’s Oncology Center of Lublin, Lublin, Poland

**Keywords:** brachytherapy, colorectal cancer, liver metastases, radiotherapy, immunotherapy

## Abstract

**Objective:**

HDR interstitial brachytherapy is one of the non-surgical methods of local treatment of liver metastases. A cancer in which local treatment of liver metastases is particularly important is colorectal cancer. The aim of the present study was to evaluate the efficacy, safety and tolerability of brachytherapy of liver metastases of colorectal cancer.

**Material and methods:**

The analysis included 270 patients with liver metastases from colorectal cancer treated between 2015 and 2022. Patients were divided into 3 groups according to indications for treatment: patients with repeat oligoprogression, induced oligoprogression and induced oligopersistence. Patients were analysed in terms of PFS and OS depending on epidemiological factors such as age and gender and clinical factors such as tumour location, type of metastases, stage, LVSI, degree of malignancy, location of extrahepatic lesions, size of metastases, number of metastases, treatment intention and dose. The degree of response to treatment and its toxicity were assessed.

**Results:**

During an average follow-up period of 16 months, the median PFS was 10 months and the OS was 17 months. 6m-, 12m-24m-PFS were 79%, 42% and 2%, respectively. and 6m-, 12m- and 24m-OS were 99%, 74% and 22%, respectively. DCR was 85%, ORR – 35%. 9% had CR, 26% PR, 50% SD, and 14% PD. The most important factors that influenced the prognosis were the intention of treatment, the degree of response to treatment, the dose administered, the presence of extrahepatic metastases, and the degree of malignancy. The toxicity of the treatment was low, and the most common side effect was pain at the injection site

**Conclusions:**

Brachytherapy is an effective and safe method of local treatment of liver metastases and should be considered especially in patients with a limited number of metastases who do not qualify for surgical treatment and the size of the metastases prevents the use of stereotactic radiotherapy.

## Introduction

Colorectal cancer (CRC) is the third most common cancer worldwide, with 1.1 million new cases reported each year. It is also the second leading cause of cancer-related deaths (1). Around 15% to 30% of patients are initially diagnosed with metastatic disease, and 20% to 50% of patients with initially locally advanced disease will develop metastases later (2). Around half of all CRC metastases are located in the liver. The primary treatment for liver metastases is surgical resection. Radical removal of metastases can achieve a 5-year survival rate of 25–40%. However, primary surgical resection is possible in only 25% of patients and secondary resection in an additional 5% (3, 4). Other methods of locally treating liver metastases include thermal techniques such as radiofrequency ablation (RFA) and laser-induced thermotherapy (LITT); transarterial procedures such as chemoembolisation (TACE) and Y-90 radioembolisation (RE); and various radiotherapy techniques, the most common of which is stereotactic radiotherapy (5–9). These methods are limited by the size, number and location of metastases, as well as the proximity of critical anatomical structures such as the stomach, intestines, bile ducts, heart and kidneys (9, 10).

An alternative to stereotactic radiotherapy is high-dose-rate (HDR) interstitial brachytherapy under continuous fluoroscopic, CT or MRI imaging control (11). Thanks to advances in imaging techniques, the miniaturisation of radioactive sources, and appropriate perioperative care aimed at the early detection and treatment of possible surgical complications, this treatment has become widely used in recent years. Due to the rapid decrease in dose with distance from the radiation source and high conformity, HDR brachytherapy treatment eliminates a number of the above-mentioned limitations. Nevertheless, there are still no large-scale studies evaluating the outcomes of HDR brachytherapy treatment for liver metastases in specific cancers, nor are there any studies determining the prognostic and predictive factors that could optimise this procedure. The following article describes the results of treating patients with liver metastases using HDR brachytherapy, based on a retrospective analysis of patients with colorectal cancer.

The study was approved by the Lublin Medical Chamber no. LIL-KB-20/2014. Written consent was obtained from each patient.

## Materials and methods

A retrospective analysis was conducted on 270 patients diagnosed with colorectal cancer and treated at the Brachytherapy Department of the Lublin Region Oncology Centre in Lublin between 2015 and 2022. The initial diagnosis of colorectal cancer was confirmed in all patients based on examination of samples taken during colonoscopy, or on the basis of histopathological examination of postoperative material if surgery was performed. The clinical stage (cTNM) was determined based on MRI or CT scans of the pelvis and chest, and the pathological stage was determined based on the surgical procedure. Liver metastases were diagnosed in all patients based on imaging tests (CT or MRI scans of the abdomen) or histopathological examination. Patients with locally advanced stages (without initially diagnosed M1 features or with a single liver metastasis) underwent surgery involving the radical removal of the tumour and regional lymphatic drainage, as well as the possible removal of liver metastases. Patients with stage T4N0M0 or T1-4N1-3M0 rectal cancer underwent preoperative sequential radiochemotherapy (a short course of radiotherapy and four to six cycles of chemotherapy based on oxaliplatin and fluoropyrimidine, or radiochemotherapy).Patients with lymph node metastases or risk factors for local recurrence were treated with adjuvant oxaliplatin-based chemotherapy with a fluoropyrimidine derivative for up to six months. Patients with inoperable metastases were treated systemically depending on the molecular status of the tumour cells. The predictors of systemic treatment in all patients were the mutation status of the RAS and BRAF genes, and DNA microsatellite instability (MSI) or disorders of the DNA mismatch repair mechanisms (dMMR). On this basis, patients were eligible for subsequent systemic treatment. First-line treatment regimens were based on fluoropyrimidine derivatives with oxaliplatin or irinotecan, combined with an EGFR inhibitor (for wild-type tumours) or a VEGFR inhibitor (for tumours with RAS/BRAF mutations). For second-line treatment, regimens based on fluoropyrimidines were used, either with oxaliplatin (if irinotecan had been used in the first line) or with irinotecan (if oxaliplatin had been used in the first line), alongside an anti-VEGFR drug. For the third line of treatment, trifluridine/tipiracil and regorafenib were used. Patients with a confirmed high level of microsatellite instability (MSI-H) or mismatch repair deficiency (dMMR) were eligible for immunotherapy with pembrolizumab.

Local treatment using HDR interstitial brachytherapy was used in three groups of patients: repeat oligoprogression (RO), induced oligoprogression (IO) and induced oligopersistence (IP), according to EORTC classification (12).

Full patient characteristics are presented in **Table 1**

**Table 1:** Patient characteristic.

Parameter	Number of patients (percentage)	Median (range)
Sex		
- men	134 (49,63%)	–
- women	136 (50,37%)	
Age	–	65 (32-87)y
≤ 50 y	21 (7,78%)	
51–70 y	171 (63,33%)	
≥70 y	78 (28,89%)	
Tumour localisation		
- rectum	135 (50%)	
- colon	135 (50%)	
• Sigmoid	43 (15,92%)	
• Descending colon	30 (11,11%)	
• Transverse colon	20 (7,41%)	
• Ascending colon	42 (15,56%)	
Number of applicators		2 (1-8)
1	50 9,77%	
2	85 16,6%	
3	73 14,25%	
4	33 6,5%	
5	23 4,5%	
6	2 0,4%	
7	1 0,2%	
8	3 0,6%	
Number of metastases		2(1-4)
1	84 16,4%	
2	111 21,68%	
3	51 20%	
4	24 4,7%	
*RAS* mutation		
*(+)*	87 (32,22%)	
(-)	173 (64,07%)	
unknown	10 (3,7%)	
*BRAF* mutation		
(+)	9 (3,5%)	
(-)	87 (32%)	
unknown	174 (64%)	
MSI-H	6 (2,22%)	
dMMR	3 (1,11%)	
none unknown	30 (11,11%) 231 (85,56%)	
Metastase type		
Metachronic	83 (30,74%)	
Synchronic	184 (68,15%)	
unknown	3 (1,11%)	
Lung metastases		
Yes	82 (30,37%)	
No	188 (69,63%)	
Peritoneum metastases/abdominal and pelvic nodese metastases		
Yes	23 (8,52%)	
No	247 (91,48%)	
Initial T		
1	3 (1,11%)	
2	30 (11,11%)	
3	190 (70,37%)	
4	47 (17,41%)	
Initial N		
0	55 (20,37%)	
1	110 (40,74%)	
2	105 (38,89%)	
Grade		
G1 G2 G3	52 (19,25%) 122 (45,19%) 12 (4,44%)	
LVSI		
(+)	61 (25,59%)	
(-)	38 (14,07)	
unknown	171 (63,33%)	
Treatment intention		
Repeat oligoprogression	127 (47,03%)	
Induced oligoprogression	75 (27,78%)	
Induced oligopersistence	68 (25,19%)	
Maximum diameter of the largest tumour		4,8 (1-10)cm
Largest average size of tumours treated during one procedure		4 (1-8)cm
≤ 4 cm	188 (69,63)	
> 4cm	82 (30,37)	
Baseline mean volume of a single tumour		65 (1-512)cm3
Baseline volume of all tumours		81 (1-1536)cm3
Dose		20 (15-25)Gy
15 Gy	54 (20%)	
20 Gy	95 (35,16%)	
25 Gy	125 (44,81%)	
D2/3 of liver		1,8Gy (0,2-4,4Gy)

MSI-H, microsatellite-instability–high; dMMR, mismatch-repair–deficient.

Inclusion criteria for patients undergoing brachytherapy were:

WHO 0-2
Tumour diameter <15cm
Number of metastases ≤ 5
Technical possibilities of application (no immediate vicinity of large vessels)
Creatinine level < 2mg/dl
HGB>8mg/dl
WBC>2000/mm3
8.NEU>1500/mm3
PLT>50,000/mm3
10.INR<1.5
11. ALT, AST, BIL total <2.5 x upper limit of normal

The exclusion criteria were

1. Resectability of the metastasis
2. Location of the tumour making it impossible to place the applicator
3. Inflammation in the abdominal cavity

### Application technique

**Figure 1:**
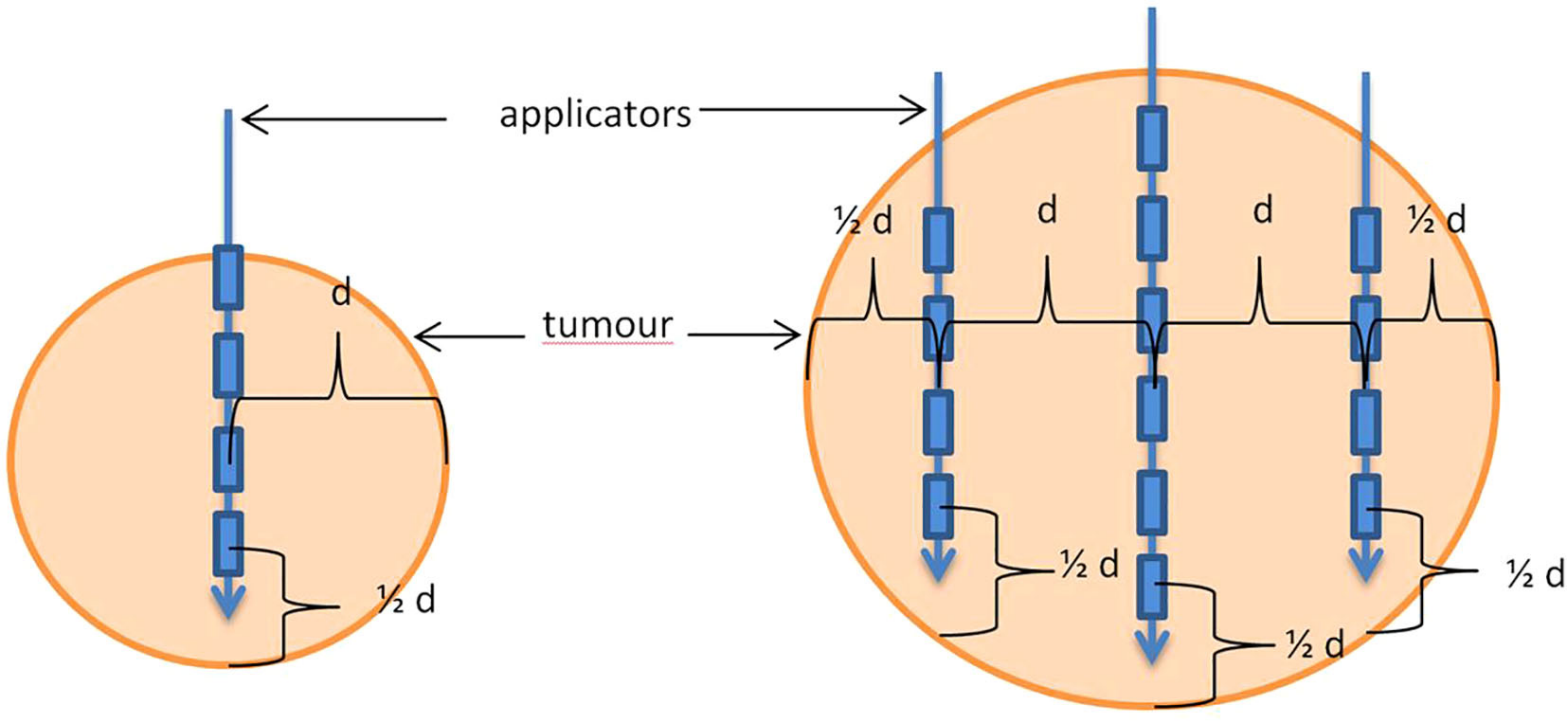
A tumour with one applicator (left) or with multiple applicators (right). “d” - maximum distance of the applicator from the edge of the tumour < 2 cm, with large tumour size above 8 cm - maximum allowed < 3 cm.

**Figure 2:**
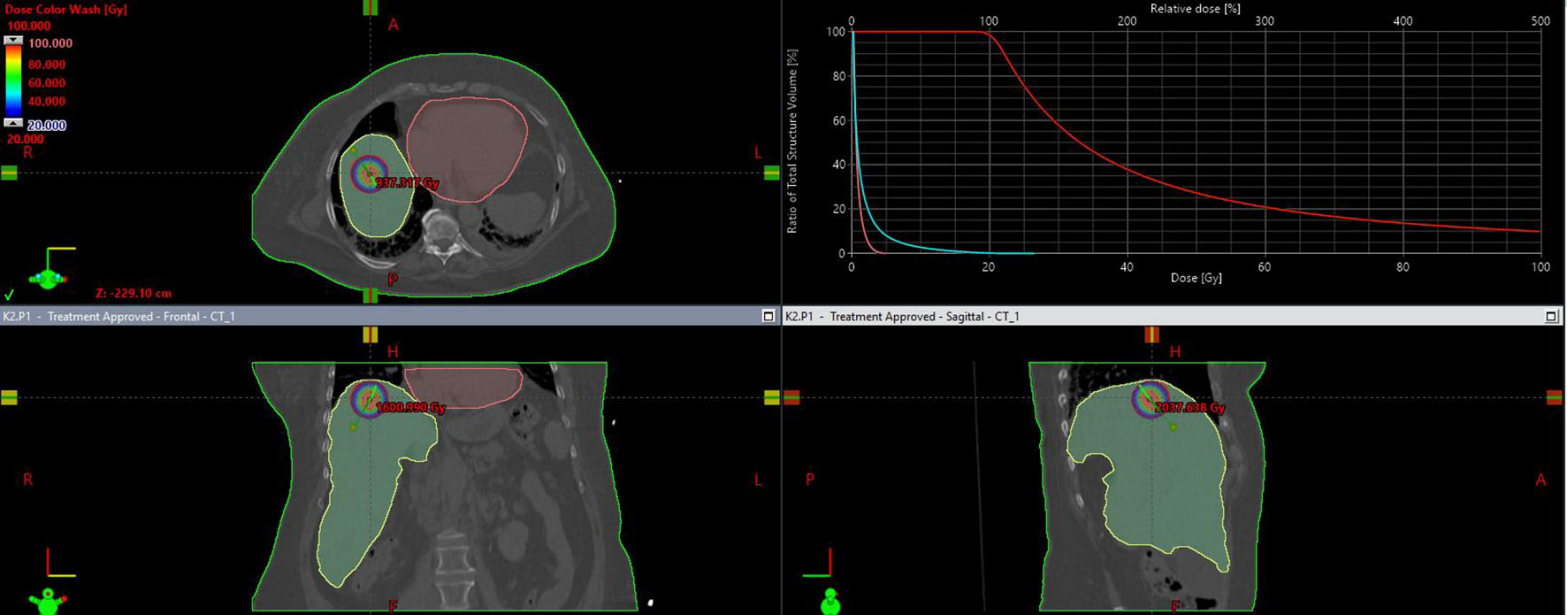
Isodose distribution on transverse, frontal, saggital plane and dose volume distribution histogram.

Patients were qualified based on their general condition, the results of current imaging and laboratory tests, the current stage of advancement, response to previous systemic treatment and the possibility of obtaining local control. 298 applications were performed. In most patients (252), applications were performed during a single procedure. The remaining patients, who required more than 4–5 applicators or had more than 3 metastases in different segments of the liver, undervent two application procedures. The application procedure was performed under general anaesthesia or local infiltration anaesthesia combined with sedation or conduction anaesthesia (intercostal block) combined with sedation. First, CT scanning with or without contrast was performed in layers at 2 mm intervals to localise the metastasis. For this purpose a 32-slice tomograph on rails with real-time fluoroscopic imaging (Somatom Siemens Germany) was used. If necessary, the CT scan was combined with other earlier imaging tests performed (MRI, PET CT). Under the control of continuous fluoroscopic CT imaging, the intercostal space was located through which access to the metastasis was as convenient as possible, i.e. it avoided large blood vessels, intestinal loops, stomach, pleura and pericardial sac. The application was carried out on the upper edge of the rib to avoid the costovascular bundle running along the lower edge of the rib. Flexible needles measuring 200 mm or 320 mm in lengh (Varian, USA) were used for application. The applicators were placed within the lesion in accordance with the Paris system rules, parallel to each other, so that the end of the applicators were positioned in the distal pole of the metastasis (due to the so-called “dead end” of the applicator, amounting to 4 mm) (**Figure 1** After the applicators were placed in the optimal position, treatment planning tomography was performed and used to inform the treatment plan. Three dose ranges (15 Gy, 20 Gy and 25 Gy) were used, depending on lesion size, doses to critical structures and treatment intention. The optimal dose distribution was considered if the specified dose was administered at a level of at least 95% of the isodose (D95), and an acceptable dose distribution was considered if the specified dose was administered at a level of at least 90% of the isodose (D90) (**Figure 2**). The main critical organ was the liver (D tolerance = D2/3 < 5Gy), as well as the stomach (D tolerance = D1cm^3^<15Gy), gall bladder (D tolerance = D1cm ^3^ max<20 Gy), intestines (D tolerance = D1cm^3^<12Gy), kidney (V7Gy<2/3 volume).

Treatment planning was performed in the Brachyvision ver. 10–12 treatment planning system (Varian USA). The treatment used a 24-channel device for remote charging of Gammamed sources (Varian USA) equipped with an Ir192 source with an average activity of 10 Ci and a diameter of 0.6 mm.

### Follow up and statistical analysis

During the post-treatment period, patients underwent regular imaging tests, including computed tomography or magnetic resonance imaging, every two to three months depending on clinical needs. The RECIST 1.0 criteria were used to evaluate the response to treatment. In some patients, MRI was also performed due to difficulties in interpreting CT images. Due to the risk of pseudoprogression in the early period (up to six weeks) after irradiation, treatment response was assessed using two subsequent CT scans, performed at least 12 weeks after brachytherapy (following two consecutive CT scans performed at an interval of at least four weeks), with no later than a five-month interval between scans. Patients were assigned to the appropriate response category according to the RECIST criteria based on changes in the size of metastases in the CT image. If an MRI scan was performed, both size change assessment and functional MRI sequences (DWI) were used to evaluate the response.

Based on the epidemiological and clinical data collected, factors influencing the prognosis of patients undergoing brachytherapy for liver metastases from colorectal cancer were identified (**Table 2**). Survival analysis was performed using the Kaplan–Meier method. Cox proportional regression analysis was used to analyse prognostic factors (dose at 90% and 100% of the isodose, the effect of chemotherapy, and the location of the primary tumour) in terms of local progression-free survival (PFS) and overall survival (OS) (13). P < 0.05 was considered to indicate a statistically significant difference. The statistical analysis was performed using Statistica ver. 13.Results

**Table 2:** Analysed epidemiological and clinical factors.

Epidemiological factors	Clinical factors
Age	Localisation
Sex	Type of metastases
	Stage (T i N)
	LVSI
	Grade (G)
	Localisation of extrahepatic sites
	Metastases sixe
	Number of metastases
	Intention of treatment
	Dose

**Figure 3:**
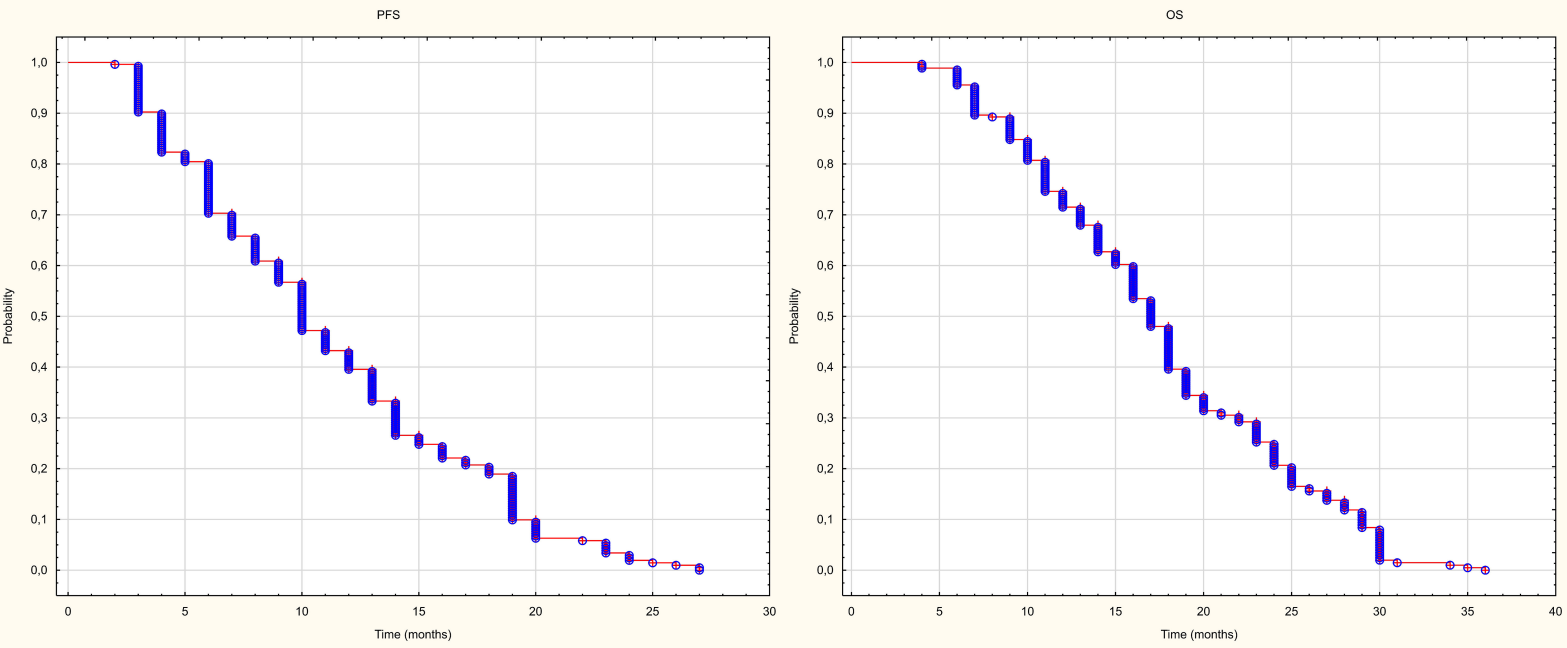
PFS and OS. Kalplan – Meier curve.

The median follow-up time for the whole group was 16 months (4–36 months). During this period, the median progression-free survival of treated metastases was 10 months. 6m-, 12m-24m-PFS were 79%, 42% and 2%, respectively. The median overall survival in the entire group was 17 months. 6m-, 12m- and 24m-OS were 99%, 74% and 22%, respectively (**Figure 3**).

**Figure 4:**
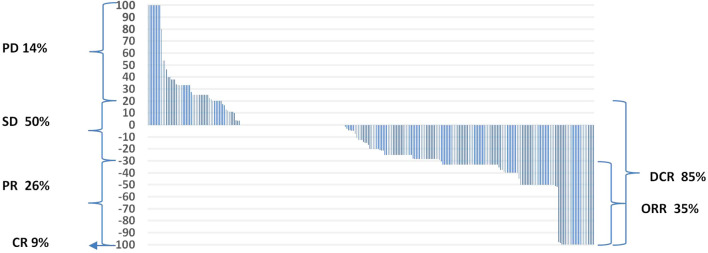
Response to treatment. DCR, disease control rate; ORR, objective response rate; PD, progression disease; SD, stabilisation disease; PR, partial response; CR, complete response.

**Figure 5:**
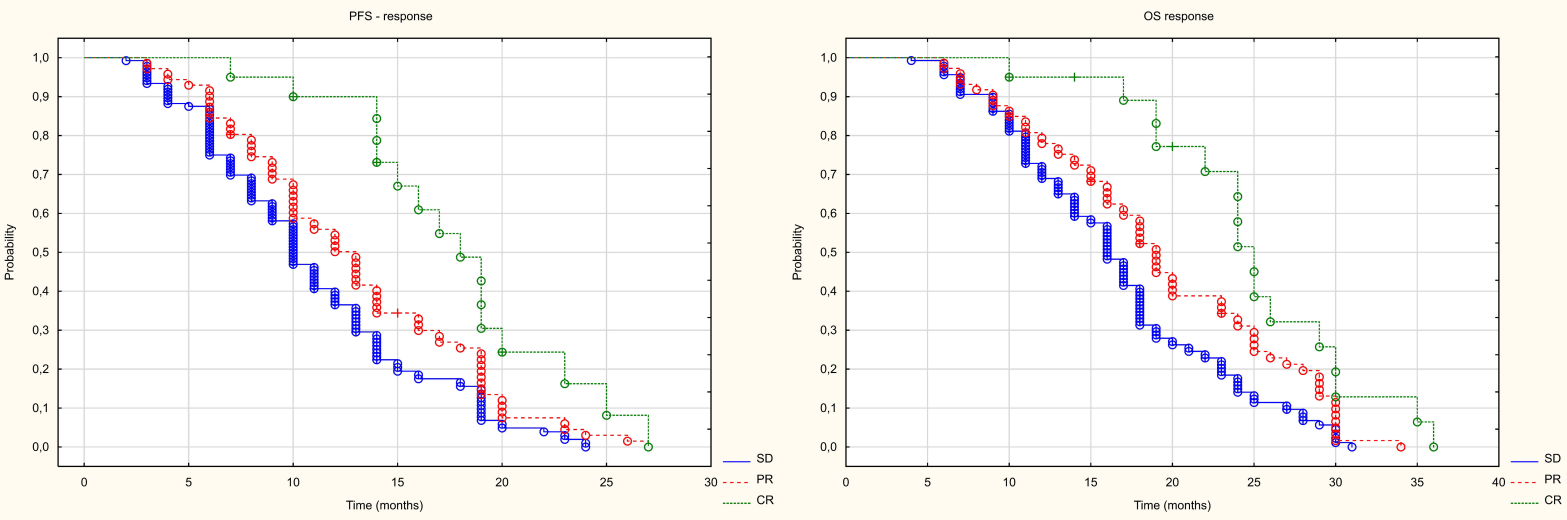
Kaplan Meier curve of PFS and OS depending on the type of response.

Of the 270 patients, the local control rate (DCR) was 85%, with an objective response rate (ORR) of 35%. 24 (9%) patients had a complete response (CR), 71 patients (26%) had a partial response, and 136 (50%) patients had stable disease (SD). 39 (14%) patients had disease progression (PD) (**Figure 4**). In patients with locally controlled disease, the RECIST response was closely correlated with PFS (median PFS in the CR groups PR and SD was 18, 12 and 10 months, respectively, p<0.001) and with OS (median OS in the CR PR and SD groups was, 25, 19 and 16 months respectively, p<0.001). The difference in PFS was statistically significant both between patients with CR and PR (HR = 0.514 (0.297-0.887), between CR and SD (HR = 0.355 (0.203-0.622), and between PR and SD (HR = 0.721 (0.533-0.974). The difference in OS was also statistically significant both between patients with CR and PR (HR = 0.524 (0.293-0.937), between CR and SD (HR = 0.372 (0.212-0.653)), and between PR and SD (HR = 0.699 (0.493-0.909). The results are presented in **Figure 5**.

**Figure 6:**
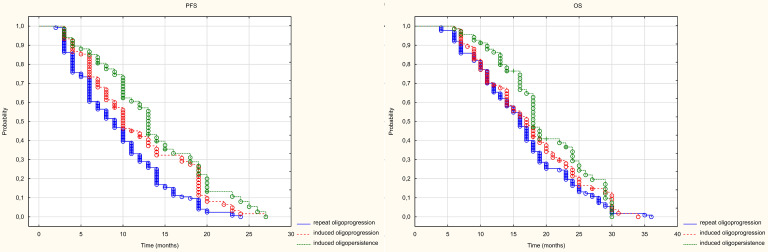
Kaplan Meier curve of PFS and OS depending on the prognosis group.

As mentioned in the previous chapter, patients were divided into three groups of patients based on their intended treatment. Statistically significant differences in PFS were found between patients with RO and those with IO (HR = 0.685 (0.506-0.927) and between those treated with RO and those with IP (HR = 0.534 (0.395-0.721). However, no statistically significant differences were found between patients treated in the groups with IO disease and those with IP disease (HR= 0.778 (0.568-1.068). The median PFS in the studied groups with RO, IO IP groups was 9, 10 and 13 months, respectively (p<0.001). Only a statistically significant difference in PFS was found between patients treated for IO and those with RO (HR= 0.686 (0.503-0.936). No statistically significant differences were found between patients treated for IP and IO (HR= 0.809 (0.575-1.139) or between those treated for IO and those with RO (HR = 0.847 (0.625-1.148). The median OS in the groups treated with RO, IO and IP was 16, 17 and 16 months, respectively (p=0.072) (**Figure 6**).

Among the epidemiological factors, age had no impact on PFS (HR 0.99, 95%CI 0.978-1.003, p=0.141) but had a marginally statistically significant impact on OS (HR 0.987, 95%CI 0.975-1.00) p =0.045. Patients in the youngest age group (50–70 years) had a longer OS than patients in the oldest age group (over 70 years), HR 0.581 95%CI 0.370-0.913). Patients’ gender also influenced the likelihood of progression. The median PFS was 12 months in women and 9 months in men (HR 0.647, 95% CI 0.491-0.853, p=0.002). The median OS differed between women and men (18 and 16 months, respectively), but these differences were not statistically significant (HR = 0.784, 95%CI 0.595-1.033), p = 0.084).

Patients were divided into two groups according to the location of the primary lesion: the rectum and the colon (ascending colon, transverse colon, descending colon and sigmoid colon). There were no statistically significant differences in PFS (p=0.84) or OS (p=0.852) between patients with tumours in different locations. Similarly, no statistically significant differences were found between patients with the tumours located on the right or left side, in terms of either PFS (HR = 0.988 95%CI (0.719-1.359) p = 0.943) or OS (HR = 0.993 95%CI (0.723) -1.391). Patients were also divided according to their initial TNM stage. There were no statistically significant differences between patients with different T characteristics, in terms of either PFS (p=0.638) or OS (p=0.774). There were no differences between patients with different N characteristics in terms of PFS (p=0.061) or OS (p=0.443). Not all patients had initially liver metastases (synchronous metastases), in some they appeared later (metachronous). Statistically significant differences were found neither in terms of PFS (HR = 0.932 95%CI (0.694-1.254) p=0.641) nor in terms of OS (HR = 0.978 95%CI (0.726-1.317) p=0.882). Lymphovascular space invasion (LVSI) identified in the surgical specimens from the primary tumour in patients with liver metastases, had no impact on PFS (HR = 0.755 95%CI (0.468-1.216) p = 0.248) or OS (HR = 0.979 95% CI (0.613-1.562), p=0.929). However, the G feature influenced both PFS and OS in patients undergoing brachytherapy for liver metastases. Patients with G3 had a worse prognosis than those with G1 or G2 in terms of PFS (HR 2.372, 95%CI (1.231-4.570) p = 0.01. There were no differences in PFS between patients with G1 and G2 (HR 0.950, 95%CI (0.679-1.330) p>0.05. The median PFS was 6 months for G3, and 11 and 10 months for G2 and G1, respectively. In addition, with regard to OS, patients with G3 exhibited a more unfavourable prognosis in comparison to those with G1 and G2 (HR 4.569, 95%CI (2.376-8.788) p<0.001. There were no differences in PFS between patients with G1 and G2 (HR 0.961, 95%CI (0.684-1.349), p>0.05. The median OS was 9 months for G3, and 17 and 19 months for G2 and G1, respectively.

**Figure 7:**
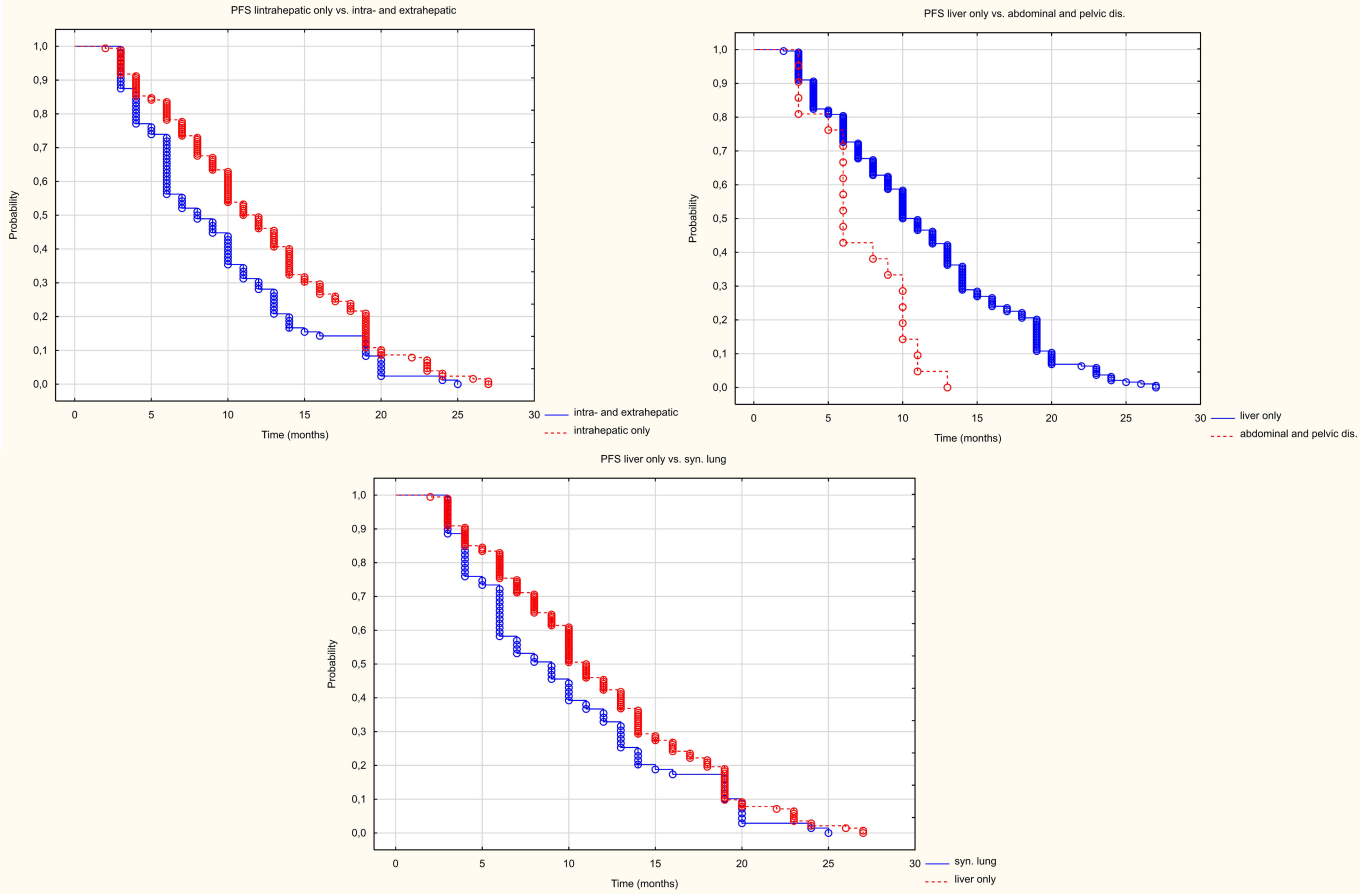
Kaplan Meier PFS curve depending on location in other organs than the liver.

Patients with metastases in the liver only had a longer progression-free survival (HR = 1.465 95%CI (1.131-1.897) p=0.004) than patients with metastases in other organs too (**Figure 7**). The median PFS in the liver-only and hepatic and extrahepatic metastases groups was 11 and 8 months, respectively. Patients with peritoneal and/or abdominal or pelvic lymph node metastases, had a statistically worse PFS than those with liver metastases only. The median PFS was 6 and 11 months, respectively (HR = 0.233 95%CI(0.116-0.467), p<0.001). However, PFS in patients with simultaneous lung metastases did not differ statistically significantly (HR = 0.746 (0.548-1.015) p = 062).

**Figure 8:**
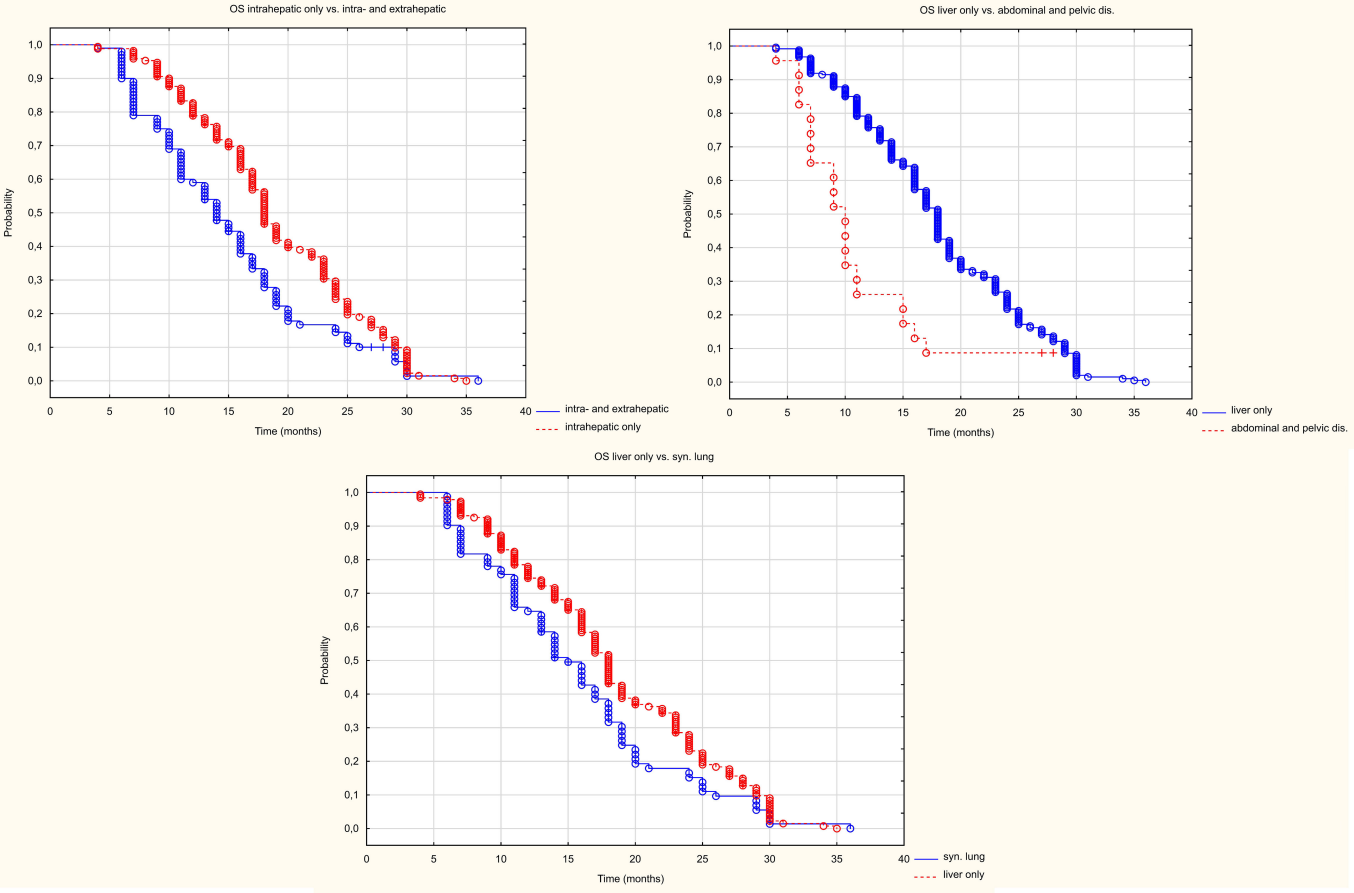
Kaplan Meier OS curve depending on location in other organs than the liver.

The presence of extrahepatic metastases worsened OS compared to patients with liver metastases only (**Figure 8**). The median OS was 18 months in patients with metastases only to the liver, compared to 14 months in patients with metastases to other organs (HR 1.52 95%CI 1.70-1.976), p=0.002). The simultaneous presence of metastases to the peritoneum and/or lymph nodes of the abdominal cavity or pelvis as well as metastases to the lungs worsened OS. Median survival was 18 m in patients with metastases only to the liver, 10 months in patients with metastases to the peritoneum and/or lymph nodes of the abdominal cavity or pelvis (HR 0.195 95%CI (0.095-0.398) p<0.001), and in patients with metastases to the lungs the median was 15 months (HR 0.703 95%CI(0.516-0.960) p=0.027).

The influence of molecular factors the outcome of treatment was also analysed. The RAS gene mutation did not affect either PFS (HR 0.764 95%CI (0.566-1.031), p=0.078) or OS (HR 0.983, 95%CI 0.733-1.317). However in the case of PFS the result was close to statistical significance (patients without RAS gene mutations had a numerically better prognosis, median PFS was 10 months in both groups, but 6-month PFS was 75% vs. 60% and 24-month PFS was 5% vs. 0%). Similarly there was no effect of MSI-H–dMMR on PFS (HR 0.594 95%CI(0.240-1.469), p=0.26) or OS (HR 0.824 95%CI(0.247-2.738), p=0.752).

**Figure 9:**
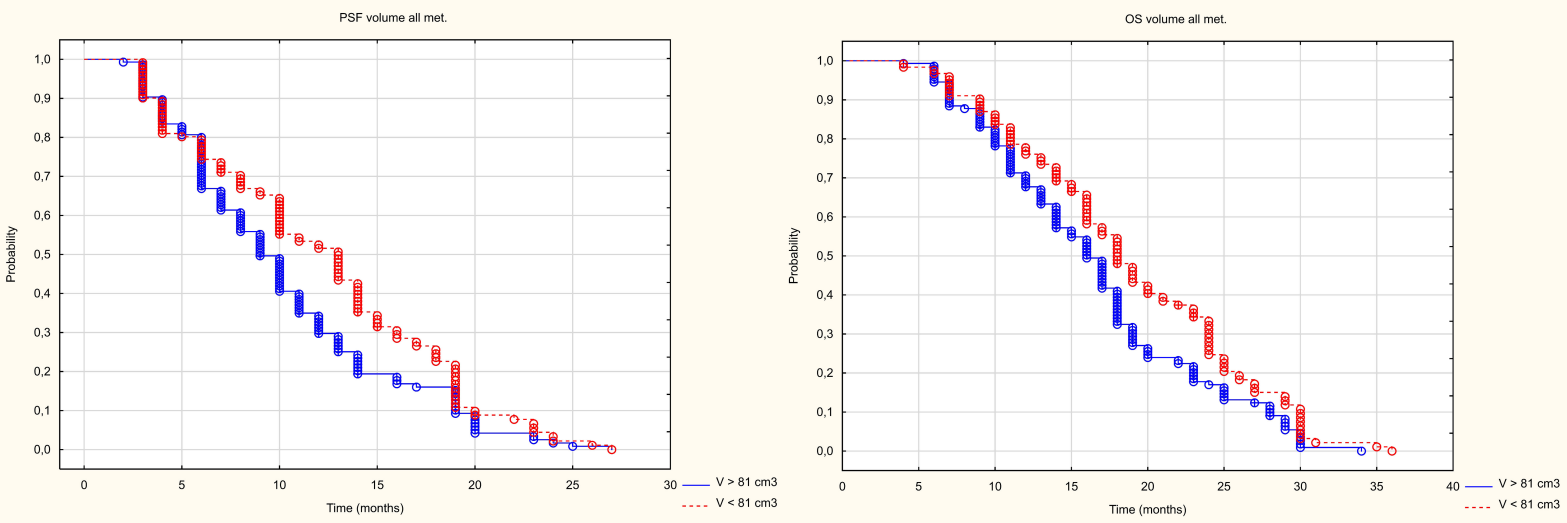
Kaplan Meier curve of PFS and OS depending on volume above and below the median.

The analysis of the number and size of metastatic lesions showed that neither the number nor average size or average volume of a single metastasis had no impact on PFS, however PFS was influenced by the volume of all metastases. Patients with a volume below the median (81cm3) experienced a longer median PFS (13m) than those with a volume above the median (PFS – 10m). The mean longest dimension and mean volume of a single metastasis had no impact on OS. However, OS was influenced by the number of metastases (patients with fewer metastases had a longer OS) and the volume of all metastases. Patients with a volume below the median (81cm3) had longer median OS (18m) than patients with a volume above the median (OS 15m) (**Figure 9**), **Table 3**.

**Table 3:** The influence of the size and number of metastases on PFS and OS.

Parameter	PFS		OS	
	HR	p	HR	p
Average longest dimension of the metastasis	1,067 (0,987-1,153)	0,104	1,031 (0,953-1,116)	0,437
Average volume of a single metastasis	1,116 (0,867-1,437)	0,393	0,968 (0,750-1,251)	0,807
Number of metastases	1,126 (0,985-1,287)	0,082	1,212 (1,056-1,389)	0,005
Volume of all metastases	1,318 (1,024-1,696)	0,032	1,362 (1,052-1,764)	0,019

**Figure 10:**
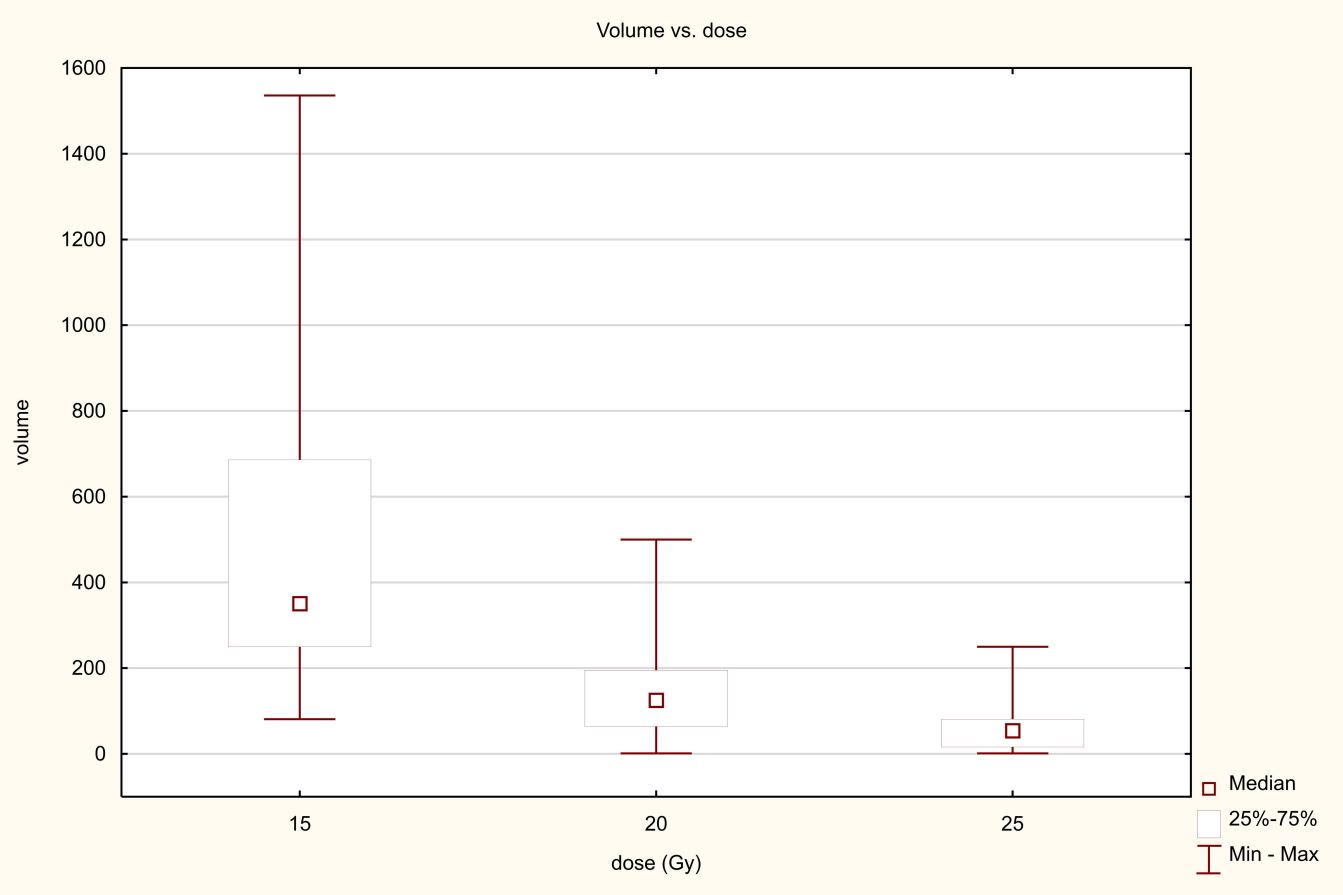
Dose-volume relationship.

**Figure 11:**
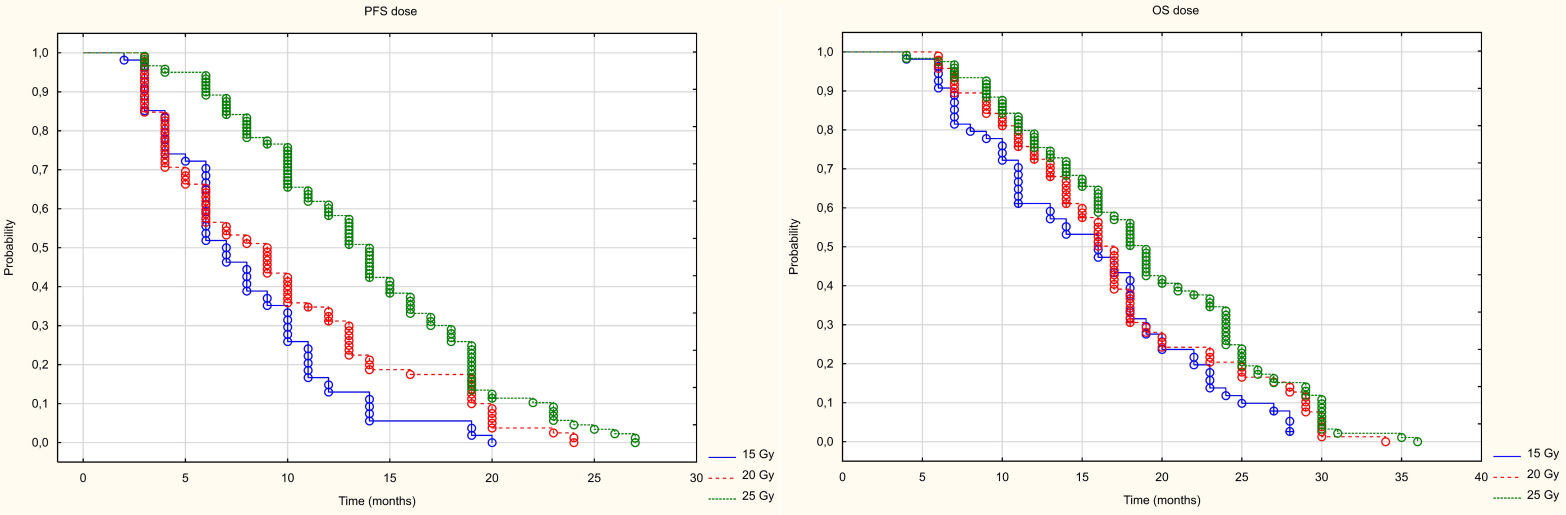
Kaplan Meier curve of PFS and OS depending on dose.

**Figure 12:**
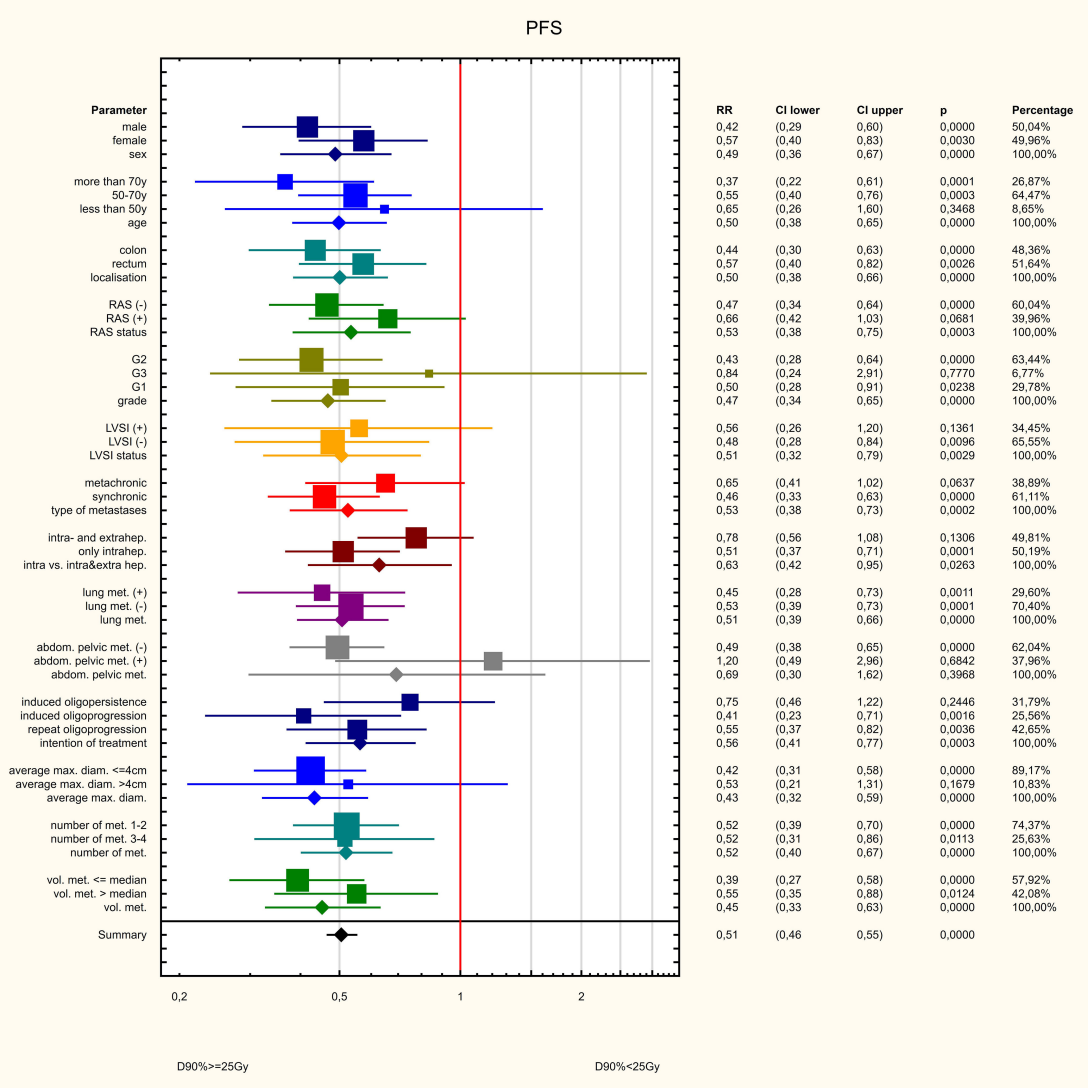
Influence of various factors on PFS.

**Figure 13:**
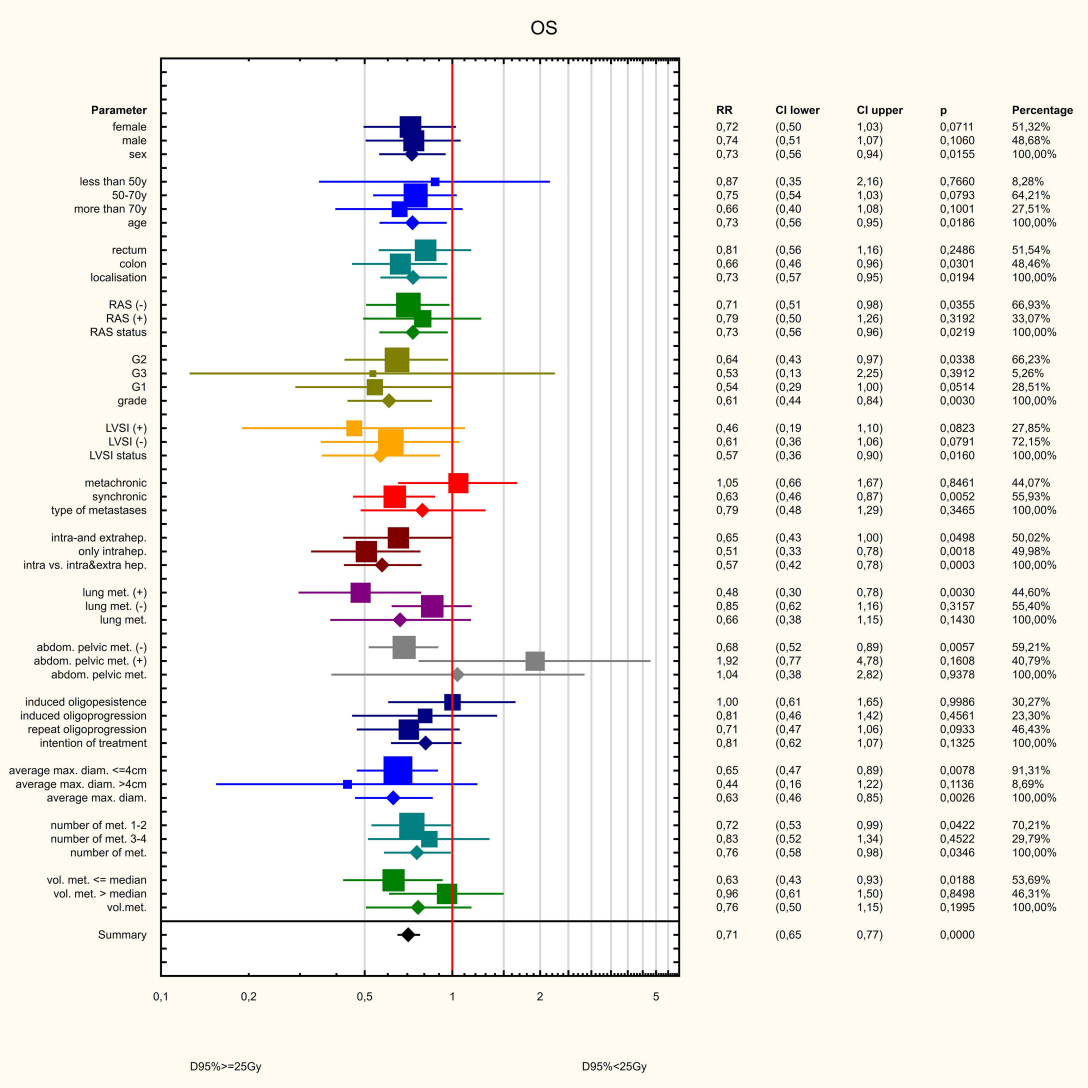
Influence of various factors on PFS.

Three dose ranges were used in the treatment: 15, 20 and 25 Gy. There was a significant correlation between the dose and the volume of the metastasis: the larger the metastasis, the lower the dose used. The differences were statistically significant (Z=82.98, p<0.001, **Figure 10**). PFS varied depending on the dose used. Statistically significant difference were found between patients who received 25 and 15Gy (HR = 0.411 (0.278-0.607) and between patients who received 20 and 15Gy (HR = 0.589 (0.443-0.781). The median PFS was 9 months in 15Gy group, 10 months in the 20 Gy group and 13 months in 25Gy group (p<0.001). There was a statistically significant difference between patients who received a dose of 25Gy and those who received a dose of 15 Gy (HR = 0.636 (0.441-0.917) but, no difference was found between patients who received 25 and 20Gy (HR = 0.788 (0.594-1.046)or between patients who received 15 and 20 Gy (0.806 (0.5469-1.190). The median OS for patients in the 15, 20 and 25 Gy groups was 16, 17 and 19 months, respectively (p=0.024) (**Figure 11**). An analysis was also performed to determine whether a higher dose (D90% ≥ 25 Gy) would be more beneficial than a lower dose (D90% < 25 Gy) (**Figure 12**). In terms of PFS, only patients with grade G3 and extrahepatic metastases in the abdomen and pelvis do not benefit from the high dose. In terms of OS, the vast majority of subgroups also benefit from a D90 dose of more than 25Gy. A lack of benefit was observed only in patients with metachronous metastases and a large volume of metastases. A smaller benefit was also found in the youngest age group. Patients with extrahepatic metastases in the abdominal cavity and pelvis benefit more from a lower dose (although this result is not statistically significant p>0.05) (**Figure 13**).

### Treatment toxicity

The treatment was generally well tolerated. The most common side effects was pain at the injection site with lasted up to 4 weeks after treatment. There were no cases of post-radiation hepatitis and only one in fourth person experienced a transient increase in the level of transaminases. In all patients, the dose to 2/3 of the healthy liver parenchyma was below 5Gy. Two serious bleeding complications, requiring urgent surgical intervention were observed in the entire group. The remaining bleeding was minor and only required conservative management. In isolated cases, clinically significant pneumothorax requiring drainage and severe mucositis with gastroscopic signs of minor bleeding appearance of radiation gastritis were observed (**Table 4**).

**Table 4:** Treatment complications.

Types of complications	CTCAE grade 1-2	CTCAE grade 3-4
Pain at the injection site	167 (59%)	10 (3%)
Increased level of transaminases	74 (26%)	4 (1%)
An increase in bilirubin levels	54 (19%)	4 (1%)
Infection	19(7%)	1 (<1%)
Bleeding	72(25%)	2 (1%)
Pneumothorax	24 (8%)	1 (<1%)
Nausea/vomiting	28 (10%)	0
Gastritis	13 (5%)	1 (<1%)

## Discussion

This study is the first large-scale analysis of treatment outcomes and prognostic and predictive factors in patients undergoing brachytherapy for liver metastases from colorectal cancer. No studies have directly compared HDR brachytherapy with the standard of radiotherapy in liver metastases - stereotactic radiotherapy. Available dosimetric analyses indicate the superiority of brachytherapy in terms of protecting of healthy liver tissue. In the study by Walter et al. (14), the average liver dose was statistically significantly lower with brachytherapy than with EBRT. Similar conclusions were drawn from the study by Hass et al. (15) - the liver volume receiving a dose of 5Gy was statistically significantly lower with brachytherapy than with stereotactic radiotherapy.

In the analysed group of patients, the median PFS was 10 months and OS – 18 months. The 6, 12 and 24 months PFS rates were 70, 40 and 2%, respectively, and the 6, 12 and 24 months PFS rates were 95.6, 71.5 and 20.7%, respectively. A relationship was also found between PFS and OS length and the degree of response in the RECIST scale. Patients with CR had a significantly better prognosis and had longer both PFS and OS. Over the last 20 years, many studies have been published on the use of brachytherapy in various locations on a relatively small groups of patients. The only prospective phase II study by Ricke et al. (16) found that LC was 80% and 53% at 6 and 9 months, respectively, while OS was 83% at 12 months. Similar treatment results have been reported in other studies using brachytherapy for liver tumours. Local control in this undifferentiated group of patients was similar, e.g. the study by Kieszko et al. (17). The 6- and 12m LC was 89 and 71%, respectively, and the 6- and 12m OS was 97 and 80% respectively. In the study by Tselis et al. (18) – the 6, 12 and 18-months LC was 89%, 73 and 63% respectively. The study by Ricke et al. (19) analysed the treatment results for patients with colorectal cancer metastases. Local control was dose-dependent with means (median) of 27.1 (25.6) months in the 15 Gy group, 31.1 (median not reached) months in the 20 Gy group, and 46.4 (median not reached) months in the 25-Gy group. The study by Colletini et al. (20), found that local tumour control after 12, 24 and 36 months was 88.3%, 81.2% and 68.4%, respectively. The median local tumour control time was 10.7 months. These results are similar to the results of the present study and a strong dose dependence is also evident. Furthermore patients receiving a dose of 25 Gy experienced significantly longer PFS than those receiving 15 or 20 Gy. Patients receiving a dose of 25 Gy had a statistically significantly longer OS than those receiving a dose of 20 Gy or 15 Gy. Analysis of our own material also allows us to conclude that a significans dose-volume relationship exist, related to the dose received by the remaining healthy part of the liver. Subgroup analysis showed that increasing the dose benefits the vast majority of patients, only patients with grade G3 and the presence of extrahepatic lesions in the abdominal cavity and pelvis do not benefit from a dose of 25 Gy or more. This is probably related to the aggressive nature of the disease and the significant impact of undifferentiated cancer on the overall prognosis. The lack of an obvious benefit in terms of OS in younger patients with metachronous metastases is probably a coincidental observation and requires further analysis. The lack of benefit from administering a higher dose to patients with large hepatic metastases is probably related to the advanced stage of the disease and greater aggressiveness, although the influence of treatment toxicity on healthy liver tissue cannot be ruled out.

Another parameter that influences prognosis is the size of the metastases. There was no statistically significant difference in prognosis between patients with different mean tumour volumes or mean longest tumour dimensions. Patients with a larger volume of metastases had shorter PFS, but this did not differ between patients with different numbers of metastases. OS was influenced by both the large number of metastases and their number. Despite the influence of the large mass of metastases, these patients also benefit from a high dose, which indicates the need to strive for the maximum possible dose also in these patients. Other studies indicate that the size of metastases influences prognosis. In the aforementioned study by Colletini et al. (20), the 12mand 24m LC were 94% and 86.8% respectively, in the group with tumours below 4 cm and 65.8 and 58.5% respectively, in the group with tumours above 4 cm. In a study on HCC (21), treated with HDR brachytherapy, the group of patients with a larger tumour volume (Me 69.25 ml) had a worse prognosis than the group with a smaller tumour volume (Me 20.41 ml) The 6-, 12- and 24-months LC was 89, 78, and 37%, respectively in the group with a larger metastatic volume and 98, 87, and 72%, respectively in the group with a smaller metastatic volume. While these results appear much more favourable than those observed in the analysed patient group, it should be noted that our study revealed much larger metastases (median volume of metastases: 81 ml). Additionally, systemic treatment had little to no effect on a large proportion of patients, as those who received it experienced progression of liver metastases.

Analysis of prognostic factors indicates that, although the prognosis for patients with extrahepatic metastases remains poor, the lack of influence of simultaneous lung metastases on progression-free survival (PFS) suggests a potential benefit of brachytherapy for patients with locally controlled lung metastases who are receiving systemic treatment. However, the impact of treatment intention on prognosis is questionable. A significant statistical difference in PFS was observed between patients in the repeat oligoprogression group and those in the induced oligoprogression and induced oligopersistence groups. A difference in OS was also observed between patients in the repeat oligoprogression group and those in the induced oligopersistence group.

Analysis of molecular factors, such as RAS gene mutations or MSI-H–dMMR, showed no influence on PFS or OS in patients undergoing brachytherapy. In endometrial cancer patients, it has been demonstrated that those with dMMR stage I to II and grades 1 and 2 who received adjuvant brachytherapy alone after surgery had a significantly higher risk of disease recurrence and worse OS (22). In our study, however, no such effect was observed in relation to dMMR features, nor with regard to PFS or OS (p = 0.26 and p = 0.752, respectively). Patients with no RAS gene mutations had a better prognosis, with the result approaching statistical significance (p=0.078). However, patients with tumours in the G3 stage had a clearly worse prognosis (p=0.01).Due to the small number of patients who underwent molecular testing, these data may not allow statistical power to be achieved, which is a limitation of the study and requires further analysis.

HDR brachytherapy treatment was found to be safe, with a low rate of side effects. In all patients, it was possible to administer a low dose to the healthy part of the liver. The rate of surgical complications was also low, decreasing over time as the therapeutic team gained experience (the last serious haemorrhagic complication occurred in 2018, and the last case of pneumothorax occurred in 2017). The tolerability of HDR brachytherapy is also confirmed by other studies. The largest of these, Mohnike et al. (23), reported a serious complication rate of 4.1% in 192 patients, which is similar to the rate reported for thermal ablation (28.29%). They indicated that the main complications were grade III–IV haemorrhage (1.46%), grade II ascites (0.29%), and gastric ulcers (0.87%), radiation-induced liver disease (0.5%), liver abscesses (1.17%), and biliary obstruction (0.9%). Minor complications included haemorrhage (3.21%), minor ascites (0.71%), and grades I and II pleural effusion (12% and 2%, respectively), as well as pneumothorax in grades I and II (1.75%). The most common symptoms reported were pain and nausea. Other smaller studies also indicate good treatment tolerance regardless of tumour location. The rate of severe complications in these studies usually did not exceed 10% (14–21, 23–25). However, in studies on the use of brachytherapy in pancreatic cancer metastases to the liver, the rate of severe complications was reported to be 15-18% (26, 27).

Despite the detailed analysis of a large group of patients who were treated using HDR brachytherapy, a local treatment method that has not yet been widely adopted, this study has several limitations. These limitations are undoubtedly the retrospective nature of the analysis, the heterogeneous patient group and the impact of systemic treatment on this group’s prognosis. Another limitation of the study is the lack of a control group. This is due to the lack of randomisation in the study. The median PFS in the study was 10 months, which, considering the different lines of treatment, is comparable to combination chemotherapy with a VEGFR or EGFR inhibitor in the first line and better than treatment in the second line (28, 29). The results of treatment with immunotherapy in the first line of treatment are significantly better, but brachytherapy was not used in the study if immunotherapy was an option. Similarly, the median OS of 17 months is higher than the median in subsequent lines of treatment in real world data analysis (30). These studies lack data on local treatments, including EBRT and brachytherapy.

## Summary

Brachytherapy for liver metastases should be considered as an alternative to surgery for patients who are not eligible for such treatment. This is particularly relevant for patients with a limited number of metastases, for whom stereotactic radiotherapy is not an option due to their size. This method is well tolerated and has low toxicity. It should also be considered for patients who are being treated symptomatically, given the clinical benefits obtained in the form of prolonged PFS and OS, with relatively good treatment tolerance.

## Data Availability

The original contributions presented in the study are included in the article/supplementary material. Further inquiries can be directed to the corresponding author.
